# Varieties of Food—Their Chemical Composition and Nutritive Value

**Published:** 1869-07

**Authors:** 


### ARTICLE VII.

### *Varieties of Food—their Chemical Composition and Nutritive Value.*

(*Concluded.*)

*Meat*—There is hardly a class of individuals, however poor, who do not make a strong effort to obtain meat. It would seem, therefore to be a necessary article of diet. In this metropolis the in-door operatives eat it to the extent of 14·8 ozs. per adult weekly; 70 per cent. of English farm laborers consume it, and to the extent of 16 ozs. per man weekly; 60 per cent of the Scotch; 30 of the Welsh; and 20 of the Irish. The Scotch, probably, have a larger allowance than the English, considering that braxy-mutton is the perquisite of the Scotch laborer; but the Welsh have only an average amount of 2½ ozs. per adult weekly; and the Irish allowance is still less.

It is difficult to obtain accurate returns of the quantity of meat consumed in London; but, if the computation of Dr. Winter be correct, it is not less than 30¾ ozs. per head weekly; or about 4⅔ ozs. per day for every man, woman and child. In Paris, according to M. Armand Husson, who has carefully collected the *octroi* returns, it is rather more than 49 ozs. per head weekly, or just 7 ozs. a day. We are not therefore, such large meat-eaters as the French.

Butcher's meat differs very much in nutritive value according to the proportions of fat and lean; and there is a strong prejudice in favor of beef as the strongest kind of meat. In reality, however, the lean of all meat is of nearly the same nutritive power, provided it is digested; but in this respect there are large differences. The flavor also varies with the nature of the animal and its mode of feeding. Pampas-pig, and indeed most wild swine, are horribly rank, but by proper feeding they become delicious. In store animals, the proportion of lean is greater than the fat, and the solid matter does not amount to more than 28 or 29 per cent.; not so however, in fat animals, for then the fat is largely in excess of the lean, and the solid matters make up about half the total weight. The tendency, indeed, of the fattening process is to substitute fat for water in the carcass; and the quality of the meat depends on the intimate intermixture of fat with the muscular tissue. All animals are not alike in their methods of depositing fat, for some put it upon the surface of the body, and others accumulate it among the viscera. The art of breeding stock is to overcome both of these tendencies, and, at the same time to produce a fat which will not melt or boil away in cooking. Oily foods have always a tendency to make soft fat.

*Lean meat* is evidently deficient of carbonaceous matter, and this is best supplied in bread or potato; but in fat meat, considering that the nutritive power of fat is twice and a half as great as that of starch or sugar, the carbonaceous matter is often in excess of the right proportions; it is remarkably so in pork, which will bear dilution with the flesh of rabbit, poultry, and veal.

The amount of bone in meat varies; it is rarely less than 8 per cent. In the neck and brisket of beef it is about 10 per cent., and in shins and legs of beef it amounts to one-third, or even half the total weight. The most economical parts are the round and thick flank, then the brisket and sticking-piece, and lastly the leg. In the case of mutton and pork, the leg is most profitable, and then the shoulder.

*Horse-flesh* is hardly known in this country, except as canine food; but on the Continent, and especially in Germany, Belgium, and Switzerland, it is regularly sold in the public markets, and is considered by many persons superior to beef. Possibly we have often eaten it on the Continent without knowing it. A *chateaubriand*, or double beef-steak of Paris, is said to be the best of horse-flesh; and no doubt the frequenters of the *restaurants* of Paris have unwittingly acquired a fondness for it, and have relished it as good beef. A story is told by a writer in the *Saturday Review* of a Frenchman who blandly remonstrated with an Englishman for his scorn of French beef. "I have," he said, "been two times in England, but I navére fine the bif supérieur to ours. I find it vary conveenient that they bring it you on little pieces of stick, for one penny, but do not find the bif supérieur." "Good Heavens!" cried the Englishman, red with astonishment, "you have been eating cat's meat." To be serious, however, I do not see why the flesh of healthy horses should not be used as human food. It has, indeed, many powerful advocates, among whom is the great naturalist, Geoffory St. Hilaire.

*Venison*, and the dark flesh of other wild animals, differs from butchers' meat in the circumstance that it is leaner, and that it contains more blood; but its nutritive power, when properly cooked, is not inferior to that of beef or mutton, and it is always more digestible.

The offal of meat constitutes about one-third of the entire weight of the slaughtered animal. It consists of the blood, the head and its contents, the tongue and brain, the heart and lungs, the abdominal viscera—as the diaphragm, the liver, spleen, pancreas, stomach, intestines, and reproductive organs, the feet, tail, and skin. In the case of the pig, the skin and head are parts of the carcass.

Nearly all these, when properly treated, are good for food. The blood of the pig is mixed with groats and fat, and converted into black pudding, which contains about 11 per cent. of nitrogenous matter. The stomach of the bullock is cleaned and boiled for tripe, which contains 13 per cent. of albumen and 16 of fat. The heart, lungs, and pancreas, which constitutes about 7 per cent. of the live weight of animals, are as nutritious as lean meat. The head, especially of the ox, makes good soup; but it requires long boiling to extract the nutriment. Boiled for eight or nine hours it will yield one-fourth of its weight in gelatine; besides which an ox-cheek will furnish about 4 pounds of good meat. Bones also contain much fat and nitrogenous matter, which they give up when broken small and boiled for many hours; 6 lbs. of bones are equal to 1 lb. of meat for nitrogen, and to nearly 2 lbs. of meat for carbon.

*Bacon* differs from fresh meat in the relatively large amount of fat and small proportion of water. It is an almost universal article of diet among the laboring classes. 74 per cent. of farm servants use it to the extent of from ¼ lb. to 2 lbs. per adult weekly. 69 per cent. of the Scotch use it, and 40 per cent. of the Irish. It is preferred to butchers' meat for many reasons—as that it goes further, especially with children, who don't generally like fat; it has more relish; it is easily cooked, and suffers less waste in cooking; besides which it is easily kept, and is always handy. Preference is nearly always given to the English bacon, notwithstanding that it is double the price of American, for the flavor is better, and it does not boil away in cooking. No doubt the inferiority of American bacon is due to the method of feeding the pigs, for they run wild and eat large quantities of acorns, and oily nuts. Good bacon should not lose more than 10 to 15 per cent. in cooking.

The peculiarity of bacon is the large amount of carbonaceous matter it contains as compared with nitrogenous. Calculated as starch, it is as 20 or 24 to 1. Hence it is that it will improve the value of substances rich in nitrogen, as eggs, veal, poultry, beans, and peas.

*Poultry* and the white meat of rabbits are not of themselves very nourishing. They contain too much nitrogenous matter and too little fat. In the case of aquatic birds, as the goose and duck, the fat is more abundant; but it contains certain flavoring matters which are not easy of digestion. The darker flesh of game is also somewhat indigestible, and requires management in its culinary treatment.

*Fish* is not a favorite article of diet with the laboring classes, unless it is salted or smoked, and then it is chiefly used for its flavoring qualities. There is a prejudice that it has no nutritive strength, and it arises, perhaps from the circumstance that it does not easily satisfy hunger, and is quickly digested; but the inhabitants of our coast use it largely as food.

The white varieties of fish, as whiting, cod, haddock, sole, plaice, flounder, and turbot, contain only about 22 per cent. of solid matter, 18 of which is nitrogenous. They want butter, therefore, to increase their nutritive value.

Mackerel, eels, and salmon, are, however, richer in fat, for the former contains about 7 per cent. and the latter 6, while the oily matter of eels amounts to nearly 14 per cent. The same is the case with the sprat, the herring and the pilchard, and with most of our fresh water fish.

All fish are in their best condition at the time of the ripening of the milt and roe, for not only are they fatter at that time but when cooked they have a better flavor, and the flesh is solid and opaque. On the other hand, when they are out of condition, the flesh is semi-gelatinous and watery.

Shell-fish of all descriptions have nearly the same nutritive values. They contain about thirteen parts of solid matter in the hundred, and this has the composition of white fish. Their digestibility varies—mussels, limpets, and whelks being rather hard of digestion, while scallops, cockles, periwinkles, lobsters, and crabs, are, perhaps, a little more easily digested, and oysters, are still more so. None of them are suited for delicate stomachs, although the poorer inhabitants on the coast eat them freely ; and vinyard snails on the Continent, and even slugs in China, have a reputation for delicacy and nutritive power.

*Eggs* contain about 26 per cent. of solid matter, 14 of which is nitrogenous, and 10½ carbonaceous or fatty. The yolk is the part which contains the fat, for it there amounts to 31 per cent., while the white of the egg, which is entirely free from fat, is the richest in nitrogen—the albumen amounting to 20·4 per cent. Altogether, however, eggs are very deficient of carbonaceous matter, for calculated as starch, it is only in the proportion of 1·75 to 1 of nitrogenous. Hence it is that eggs consort well with oil in salads, with fat bacon, and with all kinds of farinaceous matters in puddings.

*Fat* of some descriptions, as butter, lard, suet, or dripping, is universally consumed. In many cases it exists in sufficient quantity in the food, as in bacon and fat meat, but when this is not the case, it is invariably supplied from some other source. 99 per cent of farm laborers use fat of some sort—butter or dripping—to the extent of 5½ ozs. weekly per adult. It is difficult to say how much is really required by the human system, but looking at the proportion in milk, it would seem to be not less than 28 per cent. of the dry solid food. The fats in common use contain about 80 per cent. of real fatty matter, the rest being water and salt, and although butter is the fat ordinarily purchased, yet dripping is equally valuable, and so also are the vegetable fats of the tropics. Cocoa and chocolate owe their chief value as food to the fat they contain. Cocoa is composed of 50 per cent. of solid fat, called cocoa butter, and chocolate is a sweet preparation of it.

Of liquid articles of diet, beer and porter stand first in nutritive value. They contain about 9 per cent. of solid matter, 8¾ of which are sugar and gum. Their nutritive value is not, therefore, great; and yet according to Liebig, whenever beer and porter are not used, there is always a larger consumption of bread.

The nutritive functions of tea and coffee are hardly understood; for although they are largely used, and as if by an instinct craving, yet their actual nourishing power is insignificant. I shall deal further with this subject hereafter.

The last constituent of food that we have to consider is saline matter. Broadly, it may be stated that we require phosphates and sulphates of potash, lime, and magnesia, and that we also want a still larger proportion of common salt. In most cases the phosphates and sulphates are in sufficient quantity in ordinary foods; in fact Mr. Lawes found, in his experiments on the fattening of animals, that for every single part of saline matter retained in the system of the pig, there were from fourteen to fifteen parts in the food; not that the whole of this was lost, for probably it performed important functions in the process of assimilation and secretion. Common salt, however, is not present in the food to any large extent, and therefore it must be added to it.

And now, before leaving this part of the subject, let us pause to consider the vast machinery which is in operation for the supply of food to this metropolis. At the present time over three millions of people have to be fed daily; and yet so regular is the supply, that no one considers even the possibility of its failing. On the other hand, there is no redundancy; and not only does this supply regularly reach the metropolis, but it is distributed to our very doors. About 4,200 tons of fish; over 4,000 sheep; nearly 700 oxen; about 90 calves; 4,000 pigs, including bacon and hams; not less than 5,000 fowls and other kinds of poultry; besides a million or so of oysters; and eggs innumerable, with flour enough to make nearly a million quarten loaves; and vegetables, butter, and beer in proportion, are daily brought to this city. "Imagine," as Archbishop Whately says, "a head commissioner entrusted with the office of furnishing all these things regularly to the people. How would he succeed?" And yet all this goes on with the regularity and precision of machine, without Government or even municipal interference, but simply through the magical power and unfettered action of free-trade.—*Druggist Cir. & Chem. Gaz.*

